# The Successful Dentist

**Published:** 1900-10

**Authors:** E. H. Raymond


### THE SUCCESSFUL DENTIST.[1]

BY E. H. RAYMOND, D.D.S.

THE practice of dentistry to-day calls for more careful and thorough preparation, untiring research, and masterful application than ever before. The progressive development of the age almost bewilders one, and the innovations in every department of art and science show the versatility and activity of man's mind.

[1] Read before the New York Institute of Stomatology, May 1, 1900.

While dentistry as a specialty is prehistoric,—which specimens found in Etruscan tombs will prove,—there has been such a substantial advance in recent years, owing to the knowledge obtained by investigation and invention, that we are to-day behind no other profession known among men. At the end of this nineteenth century we can with pride claim the distinction of representing a profession and an art which combines in itself for its perfect accomplishment a wider range of talent and skill than many others,—an art which requires the educated eye and the cultivated taste of the artist, the wisdom and experience of the physician, the judgment and ability of the surgeon.

The importance of the teeth and their function to the health and comfort of humanity has stimulated the efforts of many of the leading and most scientific minds to devote their life-work to the preservation of these organs and the treatment of all the diseases of the oral cavity. This universal importance naturally creates a universal demand for the trained mind and hand. If the teeth were exempt from disease and disintegration, there would be few oral lesions to jeopardize the health and comfort of the race. Perfect trituration of food would insure proper digestion, and this in turn proper assimilation, preventing an overworked stomach and insuring normal processes in tissue-building throughout the body when proper food is taken.

Aside from this physiological consideration, the symmetry of the face and the pleasing expression are marred by the absence of, or by unsightly, teeth. Jewels of the rarest are secondary to them. Health and beauty depend upon them.

The high civilization of our time and the scientific preparations of food-products make it possible for every one to subsist on that which will provide for the nutrition of all the tissues of the body. There are health-food companies whose aim it is to counteract the tendency of our people to cultivate an appetite for such things as "tickle the palate," but which fail to make good blood for the nutrient supply of the various tissues of our bodies. Unfortunately, too few, from carelessness or ignorance, avail themselves of this, or provide for their growing children these substantial preparations, which nature demands to be taken into the system. Pale faces, nervous temperaments, delicate constitutions with frail teeth, and suffering, too often present themselves. Realizing, therefore, our important mission, what does this realization impose upon us?

First, an absorbing love for our chosen pursuit, and knowledge of and fitness for it.

There is one lot common to all,—the necessity to labor. Every advance that is made, whether in wealth or honor, must be made by effort; and by a gracious law of compensation, exercise develops both mind and body to greater capabilities. So surely does success follow well-directed effort, that the world long ago coined the proverb, "Labor conquers all things." On the other hand, rust not more surely tarnishes the keenest blade than does sloth and idleness impair or destroy every hope of success in any vocation.

A second motive which impels one to successful effort is the love of pre-eminence. Lurking in the breast of every one, oftentimes without the consciousness of its possession, is the feeling of gratification and pride in being pointed out to others as having attained some real or fancied superiority. The pride of success, which having once been felt and becomes so strong an incentive to activity, is but the legitimate offspring of self-esteem and self-respect, essential parts in every noble character. In its proper sphere there is no ambition purer or nobler. The eminence of the statesman, the glory of the warrior, the fame of the scholar, the reputation of the physician, the skill of the dentist, find in deserved praise not flattery, but a worthy ambition. While every one cannot be the superior of everybody else, while there are not virtues and excellencies enough to go round so that all may take an honor, it is the ability that one has to take from the material in the world, which has no distinct individual excellence, or character, and work upon it by a higher and nobler nature, and thus glorify it, which is the incentive that should enlist the earnest zeal of the dentist who hopes for distinction. It is this sentiment in working which achieves the greatest successes; which combines all the energies of mind and body in harmonious action; which takes note of neither time nor place in its absorbing interest; which takes from labor its character of toil and makes it minister to the highest enjoyment of life.

Another motive as an element essential to success is that of compensation. It might be stated as an axiom that there is one central point from which is graduated the value of every attainment, whether in literature, art, power, fame, or wealth. Like the nerves of the body, starting from the brain and pervading every minute atom of the system, so does the mysterious influence of this one element exert its power in every department of human endeavor. This material tangible and central power is money, whose ultimate representative is gold. What one action or thought is there or has there ever been that has not directly or indirectly its measurement in gold? It builds the road-bed and ties the rails of every railway; tunnels the mountains and fills up the valleys; builds the temples of religion as well as the temples of mammon; ministers to the luxuries of the rich and the necessities of the poor. It is, in short, the lever that moves the world. We claim no exemption from a law so universal, and set it down as no unworthy motive,—the hope of adequate reward as an inducement to labor.

While the field of the dentist may be circumscribed, the influence and results reach through the whole system. While the organs upon which most of his time is devoted are small, they represent the highest type of organized life, the perfected result of vital force. The structures upon which he works, the materials with which he labors, the love of blessing others, the love of benefiting himself, are all calculated to enlist an enthusiasm unknown to men in the ordinary business of life. With so much of power, by skill and delicacy of touch, to smooth the countenance distorted with pain, to quiet the nerves tingling with agony, to restore health for disease, beauty for deformity, and to change the atmosphere of decay to that of purity and sweetness,—by so strong a charm does dentistry win and hold its votaries.

Now for some very practical points,—do and do not.

The surgical treatment of the mouth is not the sole function of the dentist. Too many have failed of success because they have aspired to being surgeon-dentists, and have paid too little attention to the mechanical requirements in the office. Mechanical skill is as essential to success in dentistry as light and heat are to growth in the physical universe. No dentist ever made a name for himself without it. He may be competent as a diagnostician, but if he would—and he should—operate in such cases as antral diseases, epithelioma, pyorrhœa, etc., his skill must be pronounced, or mutilation of patient and humiliation to himself will be the result.

Let patients see at all times that we understand our business. Inspiring confidence acts as a first cause in gaining their respect as well. The patients' confidence in and respect for their dentist is the keynote to a successful career for him. A reputation once established expands in all directions, but it needs to be jealously guarded and carefully nourished by faithful service and unremitting toil. If faithful, he will never lose it.

Cultivate the grace of patience. There is nothing more trying to a man than to operate for a delicate, nervous patient, be it adult or child, but, called upon to do so, a man must exercise the best qualities of his being,—sympathy, gentleness, and a consideration for the patient to the last degree,—and use every means possible to prevent suffering. In this day of enlightenment there is no necessity for inflicting severe pain in our operations in the mouth. Little pain need be given, even in the most sensitive tissue. People will go a long distance to get into the hands of a gentle, careful operator, and they will not hesitate to speak of these qualities to others.

A man's temperament and natural traits of character, coupled with his ability, will determine the class of patients he is to minister to. His individuality has more to do with this than his environment, although the latter is important. If we are to serve the best, we must be the best and give the best. We should be deliberate in our operations, and render our best services in every case.

Be prompt in meeting engagements. A patient's time may be as valuable as an operator's, and the patient has as much right to charge for loss of time, especially if he be a business man. Avoid long sittings, unless absolutely necessary. People will dread the operating-chair if they are unduly fatigued by them. The operating-room should be the cleanest place on earth, and conspicuous for its healthful atmosphere, and the absence of the smell of drugs.

Never put into the mouth of a patient any instrument that has not been thoroughly cleansed.

Never inject cold or cool water into a sensitive cavity.

Never fill a cavity of decay of any nature without thorough antiseptic treatment.

Never go from one patient to another without washing the hands; and keep the mouth clean and healthy, so as not to be disagreeable to patients.

Never use a napkin or rubber dam that has been in the mouth of another.

Never experiment in the mouths of those who trust us, without an understanding beforehand. If we do, and fail, it will drive them away.

Never charge for a failure in any operation. Do it over until successful before rendering a bill. No man is infallible, and every one will occasionally make a mistake. Acknowledge a mistake when made. It will pay better than to try and conceal it. Never warrant an operation. We are dealing with living tissue, which is susceptible to change from many causes. Instruct patients as to the proper food for growing children; also as to the importance of keeping their mouths in condition to eat it.

Have a system for charging for services, and stick to it. The value of skilled labor cannot be measured by money, but it is best to have a system. People will know what to expect when such is the case, and it may save much controversy.

Never solicit patronage, but let the service rendered determine our ability.

Never advertise. It vitiates our ethical code, and savors of quackery.

Be careful not to speak disparagingly of a brother dentist. If he has made mistakes in diagnosis and treatment, endeavor to rectify them, without comment.

Be cheerful at all times in the office, and let the urbanity and polish of the gentleman shine out on all occasions. Keep in touch with the brethren, and strive to contribute something towards professional advancement.

